# Development of a MIRA-CRISPR/Cas12a-based nucleic acid detection system for the discrimination of *Panax ginseng* and *Panax quinquefolium*

**DOI:** 10.1016/j.jgr.2025.11.011

**Published:** 2025-11-28

**Authors:** Yanchao Yang, Dongfan Yang, Meina Shi, Zifeng Huang, Xuening Zhang, Dayuan Zheng, Tong Chu, Wenzhe Ma

**Affiliations:** a State Key Laboratory of Mechanism and Quality of Chinese Medicine & Faculty of Chinese Medicine, Macau University of Science and Technology, 999078, Macao Special Administrative Region of China; b Zhuhai MUST Science and Technology Research Institute, Macau University of Science and Technology, Hengqin Guangdong-Macao In-Depth Cooperation Zone, Guangdong, 519099, China

**Keywords:** Isothermal amplification, CRISPR/Cas12a, Lateral flow assay, Strain discrimination, *Panax ginseng*, *Panax quinquefolium*

## Abstract

**Background:**

Accurate identification of closely related herbal species is essential for ensuring the safety, efficacy, and authenticity of traditional Chinese medicine (TCM) products. *Panax ginseng* (PG) and *Panax quinquefolium* (PQ) are two widely used ginseng species with similar morphological characteristics but distinct pharmacological profiles and market values.

**Methods:**

We developed a nucleic acid detection platform that integrates multienzyme isothermal rapid amplification (MIRA) with CRISPR/Cas12a-based fluorescence or lateral flow assay (LFA) for the rapid discrimination of PG and PQ. By targeting highly divergent mitochondrial gene regions, we designed species-specific crRNAs that enabled precise identification of PG and PQ with high sensitivity and specificity.

**Results:**

By optimizing the conditions, the system can effectively distinguish PG and PQ, with a fluorescence detection limit of 10^−4^ ng/μL and a LFA detection limit of 10^−3^ ng/μL. It was also validated using 29 commercial samples including complex Chinese medicines. Compared with traditional DNA barcoding, the MIRA-CRISPR/Cas12a system demonstrated superior performance in detecting mixed or processed herbal products.

**Conclusion:**

This method offers a promising solution for point-of-care testing (POCT) in TCM quality control and provides a foundation for broader applications of CRISPR-based molecular authentication in herbal medicine.

## Graphical abstract

**Figure:**
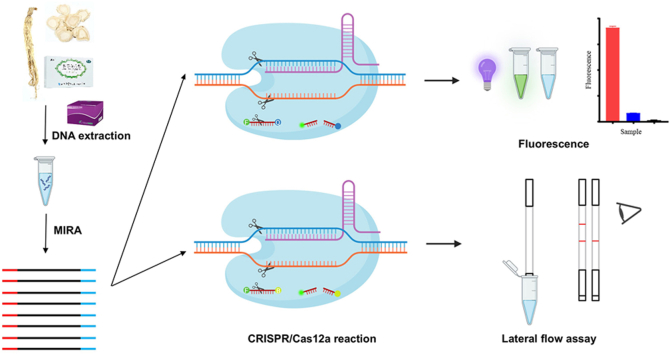

## 1 Introduction

*Panax ginseng* (PG), the dried root of *Panax ginseng* Meyer., commonly known as Oriental, Chinese, or Korean ginseng, is primarily cultivated in East Asia. *Panax quinquefolium* (PQ), the dried root of *Panax quinquefolium* L., referred to as American ginseng, is mainly grown in the United States and Canada. Both species exhibit a range of pharmacological effects, including immunomodulatory, anti-cancer, and anti-diabetic properties, which have led to the development of various derivative products such as powders, tablets, capsules, teas, and beverages [1,2]. These products are among the most widely consumed health supplements globally. However, due to their highly similar morphological characteristics, especially in processed forms, and the higher market value of PG, cases of intentional or unintentional substitution of PG with PQ have been reported [3]. To safeguard consumer rights, regulate market practices, and ensure the appropriate application of *Panax* species in traditional medicine and health products, it is crucial to establish a reliable method for on-site species discrimination.

Traditional morphological and microscopic identification methods are inadequate for distinguishing PG from PQ, particularly when the materials are processed into commercial extracts. In recent years, advanced chemical profiling techniques such as ultra-high performance liquid chromatography coupled with quadrupole time-of-flight mass spectrometry (UPLC/QTOF-MS) [4,5], high-performance liquid chromatography-mass spectrometry (HPLC-MS) [6], nuclear magnetic resonance (NMR) spectroscopy [7], and Fourier transform infrared spectroscopy (FTIR) [8] have been employed to differentiate *Panax* species. These methods primarily rely on ginsenosides profiles as distinguishing markers. However, the high degree of similarity in ginsenoside composition among *Panax* species poses significant challenges for accurate identification.

Recently, nucleic acid-based identification methods have gained increasing attention for authenticating *Panax* species. Techniques such as DNA barcoding [9], random amplified polymorphic DNA (RAPD) analysis [10], restriction fragment length polymorphism (RFLP) [11], sequence characterized amplified region (SCAR) analysis [12], next-generation sequencing (NGS) [13], and single nucleotide polymorphism (SNP) markers [14,15] have been explored. However, these methods typically require PCR amplification followed by complex readouts, necessitating professional operation and sophisticated laboratory equipment, which limits their applicability in field settings.

CRISPR-associated (Cas) nucleases, guided by CRISPR RNAs (crRNAs), have recently emerged as highly sensitive and specific platforms for nucleic acid detection [[16], [17], [18]]. In these systems, crRNA direct Cas12 or Cas13 to bind target sequences, activating collateral cleavage of a nonspecific fluorescent single-stranded DNA (ssDNA) reporter. CRISPR-Cas12/Cas13 systems have been successfully applied in the detection of pathogens such as Zika virus (ZIKV), dengue virus (DENV) [19,20], and severe acute respiratory syndrome coronavirus 2 (SARS-CoV-2) [21,22], as well as in cancer diagnostics and genetic disease screening [23,24]. Furthermore, their application scenarios have been further expanded to the food and agricultural fields, not only enabling the accurate detection of genetically modified organisms and efficient identification of food adulteration [[25], [26], [27]], but also providing extensive and practical technical support for food safety [28,29]. In the field of traditional Chinese medicine (TCM), CRISPR-based detection has been applied to identify species such as *Crocus sativus* and *Fritillaria cirrhosa*, though these applications still rely on PCR and remain confined to laboratory settings [30,31].

Multienzyme isothermal rapid amplification (MIRA) is a novel nucleic acid amplification technique that operates under constant temperature. In this method, recombinase binds to primers to form a protein/ssDNA complex, which, with the aid of accessory proteins and single-stranded binding proteins (SSBs), invades double-stranded DNA (dsDNA) to form a D-loop. DNA polymerase then rapidly amplifies the target sequence. Due to its simple primer design and high amplification efficiency, MIRA has been applied in the detection of SARS-CoV-2 [32], hepatitis B virus [33], and *Staphylococcus aureus* [34].

Lateral Flow Assay (LFA) is a point-of-care testing (POCT) technology based on antigen-antibody interactions. It utilizes chromatographic migration on a test strip, where the analyte sequentially interacts with labeled probes and immobilized antibodies, producing visible bands for result interpretation. Due to its simplicity, rapidity, and low cost, LFA is widely used in virus detection [35], food safety [36], and environmental monitoring [37].

In this study, to meet the demand for rapid on-site detection, we developed an integrated detection platform combining MIRA amplification with CRISPR/Cas12a-based LFA. This system was designed to discriminate between PG, PQ, and their related products. After comprehensive evaluation of specificity, sensitivity, and practical applicability, the method was validated through blind testing of commercial samples, demonstrating its suitability for real-world applications.

## 2 Materials and methods

### 2.1 Materials

Standard medicinal materials of *Panax ginseng* (PG) and *Panax quinquefolium* (PQ) were obtained from the National Institutes for Food and Drug Control. The Beijing Genomics Institute (Shenzhen, China) produced oligonucleotides, MIRA primers, and PCR primers for the synthesis of crRNA. The MIRA basic kit was purchased from Amp-Future Biothech (Weifang, China). LbCas12a (Cpf1), fluorescence ssDNA reporter, LFA-compatible ssDNA reporter, and CRISPR-based lateral flow strips were purchased from TOLOBIO (Shanghai, China). Life Technologies provided the RNaseOUT recombinant ribonuclease inhibitor (Carlsbad, CA, USA).

### 2.2 DNA extraction, PCR amplification, sequencing, sequence alignment and phylogenetic tree construction

Approximately 30 mg of each sample was finely ground using a household grinder. Genomic DNA was extracted using the Magnetic Plant Genomic DNA Kit (TIANGEN, Beijing, China). A Nanodrop 2000 spectrophotometer (Thermo Scientific, San Jose, CA, USA) was used to measure the concentration of DNA, which was then stored at −20 °C for additional analysis.

Universal primers were used to amplify the nuclear ribosomal DNA ITS2 region from 29 commercial samples and standard PG and PQ materials [9]. The PCR system followed the established protocols [31]. The thermal cycling conditions were referenced from the Chinese Pharmacopoeia [9].

A GelDoc XR Imaging System (Bio-Rad, Hercules, CA, USA) was used to observe the PCR products (5 μL) after they had been examined on a 1 % agarose gel at 120 V for 30 min. Sequencing was performed by the Beijing Genomics Institute. MEGA 11.0 software (Center for Evolutionary Medicine and Information, USA) was used for phylogenetic analysis and sequence alignment. The Kimura 2-Parameter model was used to build a Neighbor-Joining (NJ) tree.

### 2.3 Design and transcription of crRNAs

The crRNAs were designed using CRISPOR (https://crispor.gi.ucsc.edu/) and Cas-OFFinder (http://www.rgenome.net/cas-offinder/) for initial crRNA screening, followed by seed region mismatch analysis to eliminate off-targets [17,18]. Final crRNAs were selected based on experimental validation. Complementary oligonucleotides (crRNA-R and T7-crRNA-F) were annealed and transcribed using the T7 High Yield Transcription Kit (Thermo Scientific, San Jose, CA, USA) at 37 °C for 8 h. The RNA Clean & Concentrator™-5 (Zymo Research, Orange, CA, USA) was used to purify the transcription products after they had been treated with DNase I to eliminate any remaining DNA. The concentration of the purified RNA was measured using a NanoDrop 2000 spectrophotometer.

### 2.4 Cas12a cis- and trans-cleavage activity assays

The PCR system followed the established protocols [31], with primers *nad4*-F and *nad4*-R. The thermal profile included: 94 °C for 2 min; 40 cycles of 94 °C for 15 s, 60 °C for 15 s, and 72 °C for 25 s; and a final extension at 72 °C for 5 min. PCR products were purified using a PCR Purification Kit (Beyotime, Shanghai, China)and quantified by a Nanodrop 2000 spectrophotometer.

Cis- and trans-cleavage activities of LbCas12a were assessed in 20 μL reaction containing 1 × HOLMES buffer (TOLOBIO, Shanghai, China), 0.5 μL of Cas12a (10 μM), 1 μL of crRNA (10 μM), and 250 ng of purified PCR product. Reactions were incubated at 37 °C for 2 h and inactivated at 85 °C for 5 min. Products were analyzed on a 2 % agarose gel. For trans-cleavage assays, 1 μM of a 68 bp ssDNA substrate was added, and reactions were incubated at 37 °C for 1 h before gel analysis.

### 2.5 MIRA isothermal amplification

MIRA primers targeting the *nad4* gene were designed using Primer Premier 5 (Table S1). The lyophilized MIRA pellet was reconstituted in 29.4 μL buffer A, 4 μL of each primer (10 μM) and 9.1 μL nuclease-free water. The MIRA mixture was divided into two tubes, and 2.5 μL DNA template and 1.25 μL buffer B were added to initiate amplification. The reactions were conducted at 39 °C for 30 min. Products were analyzed on a 2 % agarose gel and visualized using the GelDoc XR Imaging System.

### 2.6 CRISPR/Cas12a detection system

The Cas12a-mediated detection assay was conducted in 20 μL reaction containing 1 × HOLMES buffer (TOLOBIO, Shanghai, China), 0.1 μL of Cas12a (10 μM), 0.1 μL of crRNA (10 μM), 0.1 μL of fluorescence ssDNA reporter (10 μM), 0.1 μL of RNaseOUT recombinant ribonuclease inhibitor (40 U/μL), and 1.0 μL of MIRA product. Reactions without DNA served as negative controls. The fluorescence was measured using a SpectraMax iD5 microplate reader (excitation: 485 nm; emission: 525 nm) after 30-min incubation at 37 °C. For visual detection, reactions using LFA-compatible ssDNA reporter were analyzed with LFA strips. After mixing 20 μL reaction product with 30 μL nuclease-free water, strips were inserted and results were read after 5–10 min. Band intensity was quantified using ImageJ, with the ratio of upper to lower band intensity used for analysis.

### 2.7 Optimization of MIRA-CRISPR/Cas12a assays

To optimize assay conditions, various MIRA primer sets, reaction temperatures, Cas12a/crRNA ribonucleoprotein (RNP) concentrations, and ssDNA reporter concentrations were evaluated. Amplification products were analyzed via agarose gel electrophoresis, while fluorescence and LFA results were used to determine optimal conditions.

### 2.8 Sensitivity, specificity and detectability of the MIRA-CRISPR/Cas12a system

To assess specificity, each crRNA was tested against PG-SMM, PQ-SMM, and a no-template control (NTC). Sensitivity was evaluated using serial 10-fold dilutions of DNA templates (10^−7^ to 10^−1^ ng/μL). Meanwhile, mixed samples spiked with PG-SMM and PQ-SMM DNA at varying proportions (100 %, 50 %, 20 %, 10 %, 1 %, 0.1 %, 0 %) were used to assess the detectability. The cutoff value was defined as the mean background signal (n = 3) plus three times the standard deviation (B + 3SD) [38,39].

### 2.9 Real sample testing and method validation

To validate the practical applicability of the MIRA-CRISPR/Cas12a system, 29 commercial samples from various brands and sources were collected. DNA was extracted and analyzed using the established method. Results were verified by DNA barcoding.

## 3 Results

### 3.1 DNA barcoding analysis of PG and PQ standard medicinal materials

Standard samples of *Panax ginseng* (PG) and *Panax quinquefolium* (PQ) were obtained from the National Institutes for Food and Drug Control. To verify their authenticity, DNA barcoding was performed using the ITS2 region of ribosomal DNA, as previously described [9]. PCR amplification yielded 497 bp products for PG standard medicinal material (PG-SMM), PQ standard medicinal material (PQ-SMM), and their 1:1 mixture (Supplemental Fig. S1A). Sequencing results revealed that PG and PQ ITS2 sequences shared high homology, differing by only two SNPs. The mixed sample exhibited double peaks at both SNP loci (Supplemental Fig. S1B). Sequence alignment confirmed 100 % identity with reference sequences, validating the authenticity of the standard materials used in this study (Supplemental Fig. S1C).

### 3.2 Design specific crRNAs for PG and PQ

**Fig. 1:**
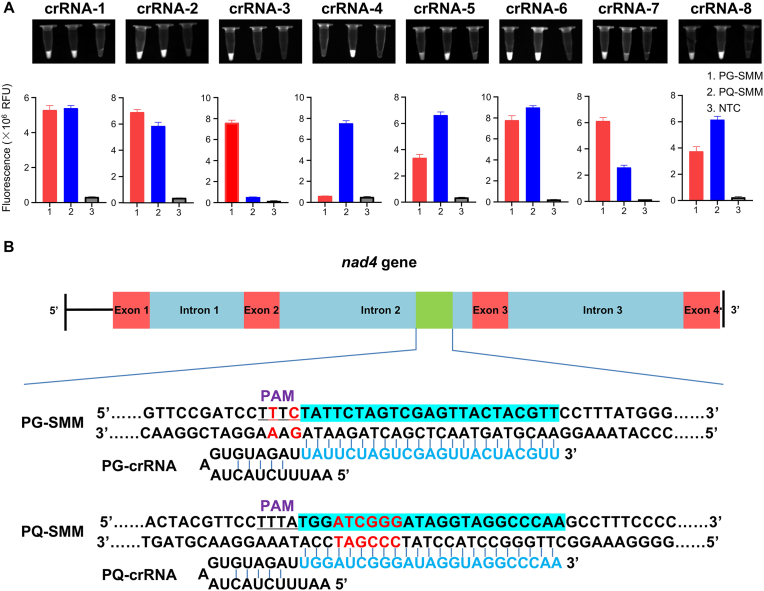
**Design specific crRNAs for PG and PQ.** A. The images of fluorescence detection and fluorescence signals of CRISPR/Cas12a-based detection of PG-SMM and PQ-SMM with 8 different crRNAs (n = 3 technical replicates; bars represent the mean ± SD; All experiments were performed at least three biologically independent times with similar results). B. Gene map of the *Panax nad4* DNA fragment with detailed sequence information of the designed crRNAs.

Due to the high sequence similarity of ITS2 between PG and PQ (Supplemental Fig. S1C), it was not feasible to design species-specific crRNAs based on this region. Therefore, we turned to the mitochondrial genome, which typically exhibits greater sequence divergence, particularly in intronic regions. Four crRNAs targeting the *nad2*, *nad4*, and *nad5* genes were designed for each species. Among them, a pair targeting the *nad4* intron 2 (crRNA-3 and crRNA-4) demonstrated excellent specificity under fluorescence detection (Fig. 1A). Multiple mismatches in the "seed regions" contributed to their high discrimination power (Fig. 1B). These were designated as “PG-crRNA” and “PQ-crRNA”, respectively.

To validate the cleavage activity of the Cas12a/crRNA ribonucleoprotein (RNP) complex, we performed both cis- and trans-cleavage assays. In the cis-cleavage assay, the full-length double-stranded DNA (∼400 bp) was cleaved into expected fragments (∼300 bp), confirming site-specific cleavage (Supplemental Fig. S2A). In the trans-cleavage assay, degradation of the ssDNA reporter occurred only when Cas12a, crRNA, and target DNA were all present, indicating that trans-cleavage activity was strictly target-dependent (Supplemental Fig. S2B).

### 3.3 Optimization of MIRA-CRISPR/Cas12a assays

**Fig. 2:**
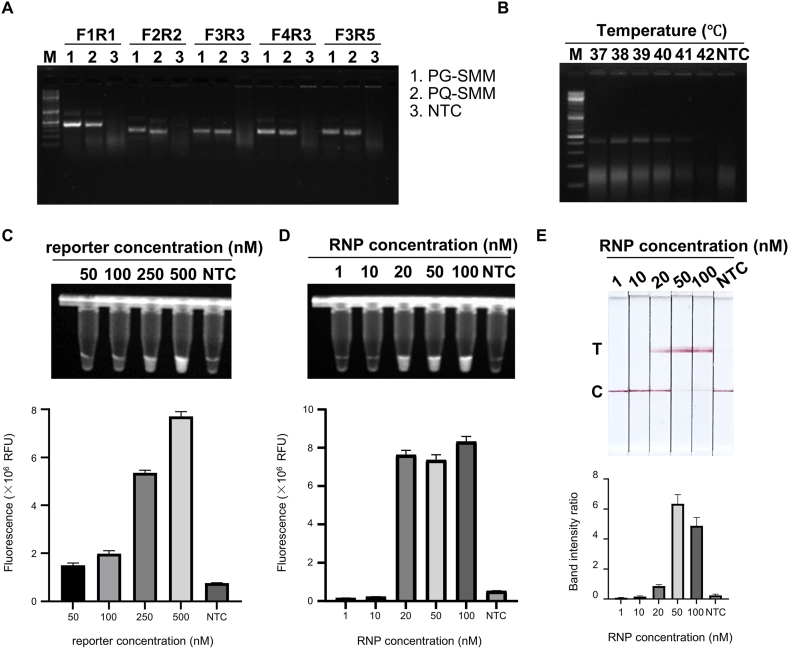
**Optimization of MIRA-CRISPR/Cas12a assays.** A. Agarose gel image for comparison of 5 pairs of MIRA primer. M: Marker. B. Agarose gel image of MIRA temperature gradient. M: Marker. C. The images of fluorescence detection and fluorescence signals of MIRA-CRISPR/Cas12a-based detection with a reporter concentration gradient. D. The images of fluorescence detection and fluorescence signals of MIRA-CRISPR/Cas12a-based detection with a Cas12a/crRNA ribonucleoprotein (RNP) concentration gradient. E. The images of lateral flow assay (LFA) detection and band intensity ratios of MIRA-CRISPR/Cas12a-based detection with a RNP concentration gradient. C: control line T: test line. All data are presented as the mean ± SD (n = 3 technical replicates). All experiments were performed at least three biologically independent times with similar results.

MIRA was selected over PCR due to its isothermal nature, eliminating the need for thermal cyclers and making it more suitable for field applications. A series of primer sets were screened, and primer pair 1 produced the most robust amplification (Fig. 2A). Temperature optimization revealed that 39 °C yielded the best amplification efficiency (Fig. 2B).

The concentration of the ssDNA reporter significantly influenced signal intensity and background noise. A final concentration of 500 nM provided the strongest fluorescence signal (Fig. 2C). RNP concentration also affected cleavage efficiency. Fluorescence peaked at RNP concentrations between 20 and 100 nM, with 50 nM producing the most distinct test (T) line on LFA strips (Fig. 2D and E). Therefore, 50 nM was selected as the optimal RNP concentration.

### 3.4 Sensitivity, specificity and detectability of the MIRA-CRISPR/Cas12a system

**Fig. 3:**
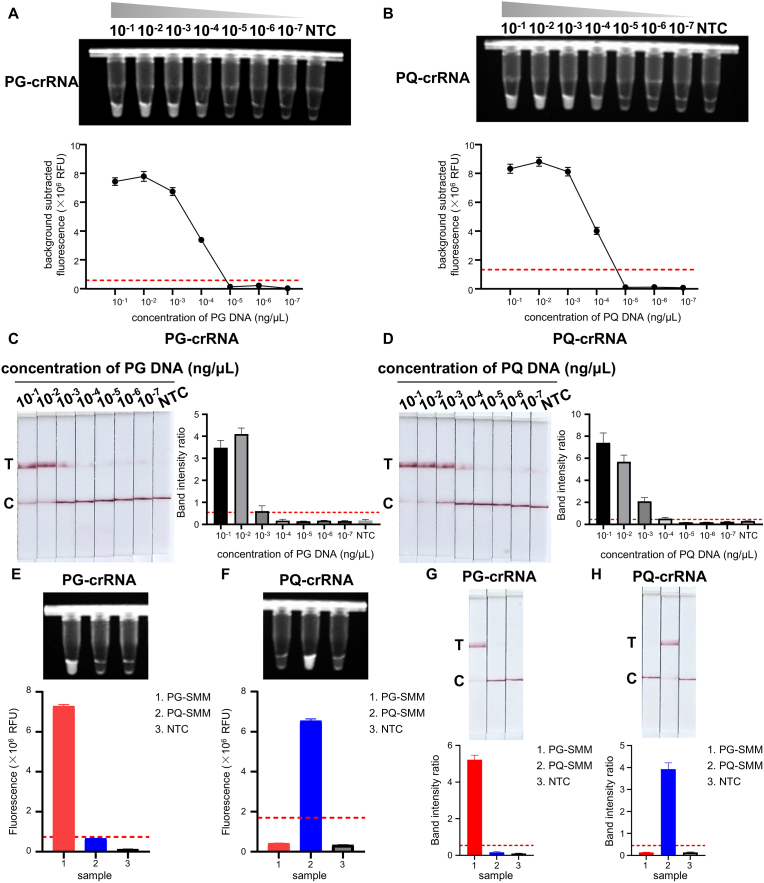
**Sensitivity and Specificity assessment based on MIRA-CRISPR/Cas12a detection of PG-SMM and PQ-SMM.** A-B. The images of fluorescence detection and fluorescence signals of MIRA-CRISPR/Cas12a-based detection of 10-fold diluted DNA templates of PG-SMM and PQ-SMM with PG-crRNA and PQ-crRNA (the cutoff fluorescence intensity values for PG-crRNA and for PQ-crRNA at 7.4 × 10^5^ and 1.7 × 10^6^ RFU for positive samples). C-D. The images of lateral flow assay (LFA) detection and band intensity ratios of MIRA-CRISPR/Cas12a-based detection of 10-fold diluted DNA templates of PG-SMM and PQ-SMM with PG-crRNA and PQ-crRNA (the cutoff LFA band intensity ratios for PG-crRNA and for PQ-crRNA at 0.54 and 0.45 for positive samples). C: control line T: test line. E-F. The images of fluorescence detection and fluorescence signals of MIRA-CRISPR/Cas12a-based detection of PG-SMM and PQ-SMM with PG-crRNA and PQ-crRNA. G-H. The images of lateral flow assay (LFA) detection and band intensity ratios of MIRA-CRISPR/Cas12a-based detection PG-SMM and PQ-SMM with PG-crRNA and PQ-crRNA. C: control line T: test line. All data are presented as the mean ± SD (n = 3 technical replicates). All experiments were performed at least three biologically independent times with similar results.

To evaluate sensitivity, serial dilutions of PG and PQ DNA templates (10^−7^ to 10^−1^ ng/μL) were amplified by MIRA (Supplemental Fig. S3A and S3B). Using fluorescence detection, the cutoff values for PG-crRNA and for PQ-crRNA were calculated as 7.4 × 10^5^ and 1.7 × 10^6^ RFU, respectively. Therefore, the detection limit was 10^−4^ ng/μL, representing a 100-fold increase in sensitivity compared to MIRA alone (Fig. 3A and B). When LFA strips were used for detection, the red test line gradually faded with decreasing DNA concentration. For PG-crRNA, the test line became indistinct at 10^−3^ ng/μL, while PQ-crRNA maintained visibility down to 10^−4^ ng/μL, the cutoff LFA band intensity ratios for PG-crRNA and for PQ-crRNA were calculated as 0.54 and 0.45 (Fig. 3C and D).

Specificity tests showed that PG-crRNA only detected PG DNA, and PQ-crRNA only detected PQ DNA, with no cross-reactivity or background signal in the no-template control (NTC) (Fig. 3E and F). Fluorescence and LFA results were consistent, confirming the high specificity of the system (Fig. 3G and H).

Furthermore, we verified the detectability of this method using a series of mixed samples containing varying PG and PQ DNA proportions, designed to simulate the adulteration levels encountered in practical applications. Fluorescence and LFA detection revealed clear signals at 0.1 % and 1 % PG/PQ DNA, respectively (Fig. S4C and S4D), indicating that our method is capable of detecting adulteration levels as low as 1 %.

### 3.5 Detection of commercial samples

**Fig. 4:**
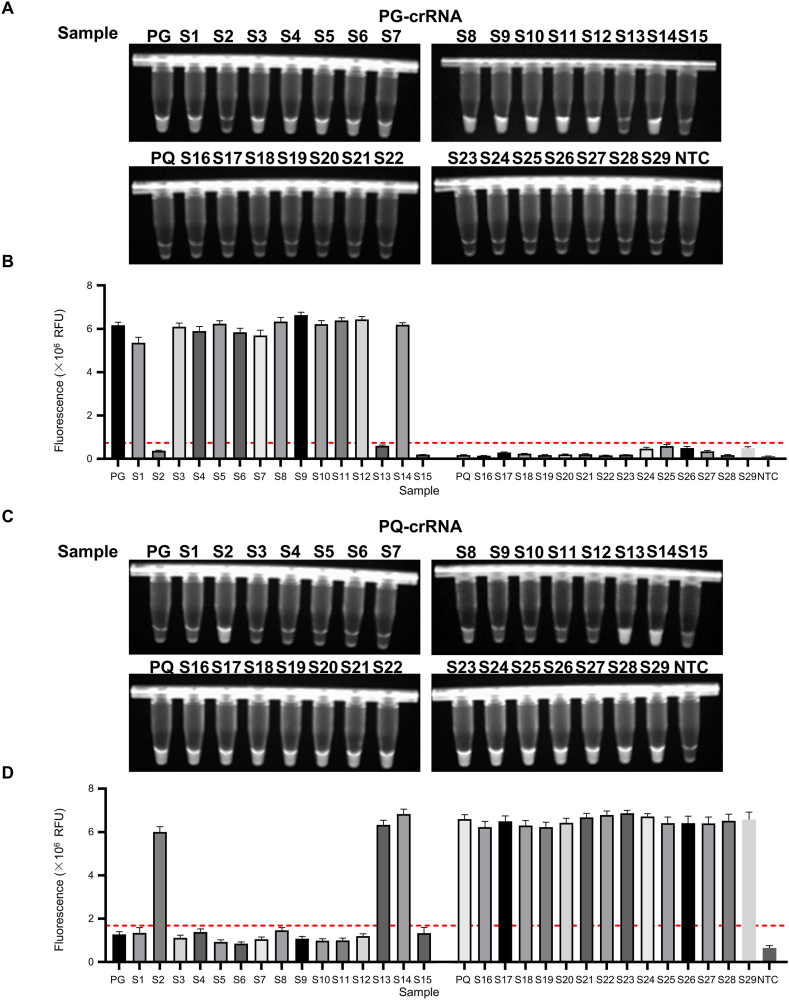
**Fluorescence detection of commercial samples based on MIRA-CRISPR/Cas12a.** A-B. The images of fluorescence detection and fluorescence signals from the MIRA-CRISPR/Cas12a-based nucleic acid detection of 29 samples, PG-SMM, PQ-SMM and NTC using PG-crRNA (the cutoff fluorescence intensity values for PG-crRNA at 7.4 × 10^5^ RFU for positive samples). PG: PG-SMM PQ: PQ-SMM. C-D. The images of fluorescence detection and fluorescence signals from the MIRA-CRISPR/Cas12a-based nucleic acid detection of 29 samples, PG-SMM, PQ-SMM and NTC using PQ-crRNA (the cutoff fluorescence intensity values for PQ-crRNA at 1.7 × 10^6^ RFU for positive samples). PG: PG-SMM PQ: PQ-SMM. All data are presented as the mean ± SD (n = 3 technical replicates). All experiments were performed at least three biologically independent times with similar results.

**Fig. 5:**
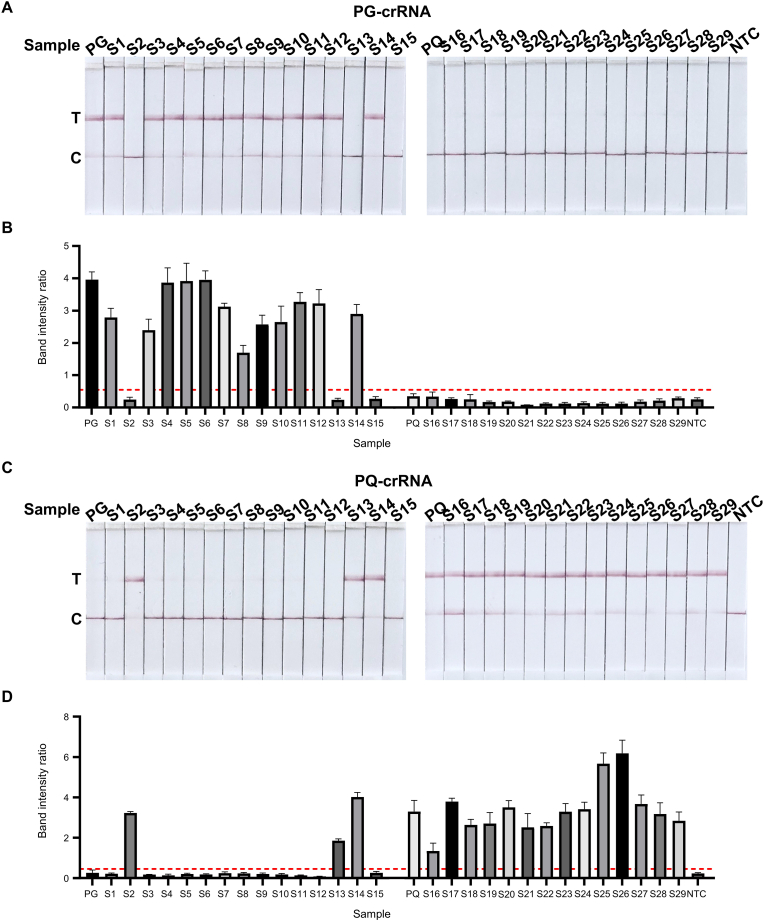
**LFA detection of commercial samples based on MIRA-CRISPR/Cas12a.** A-B. The images of lateral flow assay (LFA) detection and band intensity ratios from the MIRA-CRISPR/Cas12a-based nucleic acid detection of 29 samples, PG-SMM, PQ-SMM and NTC using PG-crRNA (the cutoff LFA band intensity ratios for PG-crRNA at 0.54 for positive samples). PG: PG-SMM PQ: PQ-SMM C: control line T: test line. C-D. The images of lateral flow assay (LFA) detection and band intensity ratios from the MIRA-CRISPR/Cas12a-based nucleic acid detection of 29 samples, PG-SMM, PQ-SMM and NTC using PQ-crRNA (the cutoff LFA band intensity ratios for PQ-crRNA at 0.45 for positive samples). PG: PG-SMM PQ: PQ-SMM C: control line T: test line. n = 3 technical replicates; bars represent the mean ± SD; All experiments were performed at least three biologically independent times with similar results.

A total of 29 commercial *Panax* samples were collected from pharmacies and online platforms. These included: S1–S9 (PG medicinal materials), S10-S12 (PG herbal pieces), S13-S15 (Chinese patent medicines containing PG), S16–S18 (PQ medicinal materials), S19-S28 (PQ herbal pieces), and S29 (a PQ-containing patent medicine) (Table S2 and S4). Most samples (25/29) were consistent with their labeling. However, S2 and S13, labeled as PG, tested negative for PG and positive for PQ in both fluorescence (Fig. 4A-D) and LFA detection (Fig. 5A-D). Interestingly, S14 tested positive for both PG and PQ, while S15 was negative for both (Fig. 4A-D and Fig. 5A-D).

**Fig. 6:**
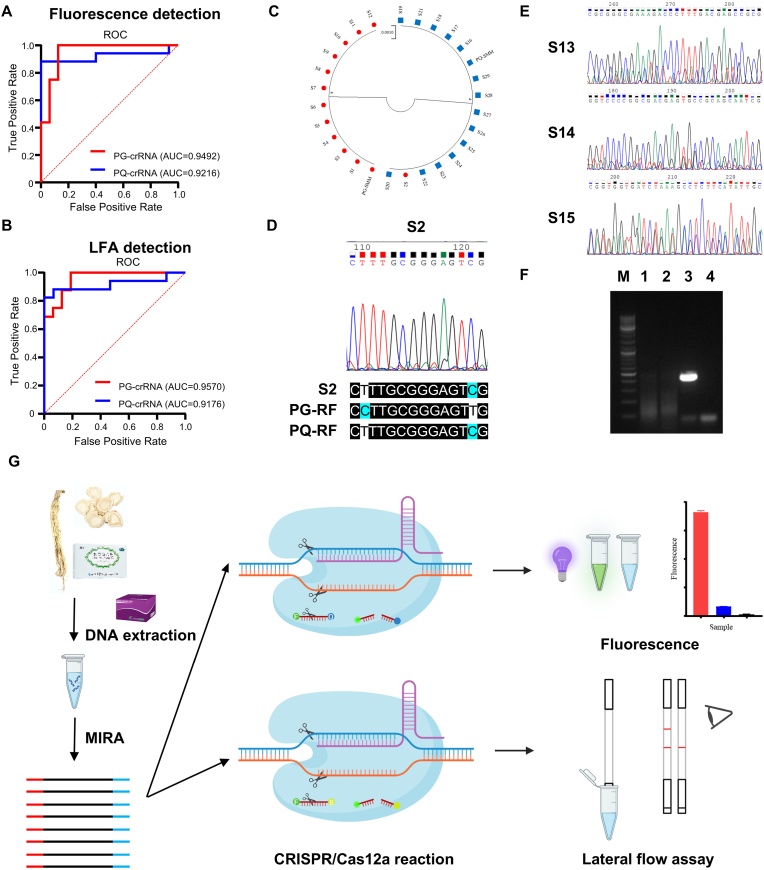
**ROC-AUC analysis, DNA barcoding analysis for verification and the flow chart of the MIRA-CRISPR/Cas12a-based nucleic acid detection.** A-B. ROC-AUC analysis of real Samples via fluorescence and LFA detection (n = 32). C. Sequence alignment and NJ tree based on the 26 commercial samples, PG-SMM and PQ-SMM sequences. (Red dots represent PG, Blue squares represent PQ). D. The partial sequencing chromatogram of S2 and the partial sequence from the alignment of ITS2 sequence of S2 with PG reference sequence (PG-RF) and PQ reference sequence (PQ-RF). E. The partial sequencing chromatogram of S13, S14 and S15. F. Agarose gel image of MIRA and PCR products from S15. M: Marker 1: MIRA products from S15 2: MIRA products from NTC 3: PCR products from S15 4: PCR products from NTC. G. The flow chart of MIRA-CRISPR/Cas12a-based nucleic acid detection process (image created using the BioRender application).

Subsequently, we performed ROC-AUC analysis on real samples, which demonstrates that the MIRA-CRISPR/Cas12a system exhibits excellent discriminatory performance (Fig. 6A and B). To further interpret these results, DNA barcoding was performed on all 29 samples. The 26 pure medicinal materials matched their reference sequences (Fig. 6C and Table S3). S2 was confirmed to be mislabeled and should be classified as PQ (Fig. 6D). S13, S14, and S15 showed multiple peaks, consistent with their nature as multiple-herbal patent medicines (Fig. 6E). CRISPR detection revealed that S13 was mislabeled (containing PQ instead of PG), S14 contained both PG and PQ, and S15 yielded no signal—likely due to lower amplification efficiency of MIRA compared to PCR (Fig. 6F). These findings confirm the accuracy and practical application of the MIRA-CRISPR/Cas12a system for distinguishing PG and PQ in commercial products.

## 4 Discussion

Although there are subtle morphological differences between PG and PQ, such as the removal of fibrous roots in PQ, these distinctions become nearly indistinguishable when the materials are processed into herbal pieces or powders (Supplemental Fig. S4). In recent years, molecular identification of *Panax* species has attracted increasing attention, with DNA barcoding emerging as a widely adopted approach [40]. However, DNA barcoding is primarily effective for pure medicinal materials.

In cases involving mixed or contaminated samples, overlapping PCR products can result in ambiguous sequencing peaks, severely compromising sequence interpretation. This limitation is particularly evident in traditional Chinese patent medicines, where DNA fragments from multiple herbal components are co-amplified, rendering conventional barcoding ineffective. For example, samples S13, S14, and S15 exhibited multiple peaks due to their complex formulations (Fig. 6E). In contrast, the MIRA-CRISPR/Cas12a-based detection system demonstrated a unique advantage in identifying specific components within multi-herb formulations, as exemplified by the accurate detection of PQ in S13.

In this study, mitochondrial genes were selected as the target sequences for CRISPR detection instead of commonly used ITS2 or chloroplast genes. This choice was based on the higher sequence divergence typically observed in mitochondrial intronic regions among closely related species. Although plant mitochondrial genomes evolve slowly overall, they exhibit frequent recombination and localized hypervariability, which can accumulate substantial interspecies differences [14,41]. These characteristics provide ideal conditions for designing highly specific crRNAs. In contrast, ITS2 and chloroplast genes, while widely used as DNA barcodes, often display high conservation among closely related species, limiting their utility for designing discriminatory crRNAs.

Another limitation of DNA barcoding is its reliance on laboratory infrastructure. Both PCR amplification and sequencing require specialized equipment and trained personnel. In contrast, the MIRA-CRISPR/Cas12a system developed in this study enables visual detection via ultraviolet light or lateral flow strips (Fig. 6G). For DNA extraction, specific adsorption of DNA by magnetic beads is utilized, and impurities such as polysaccharides and tannins are separated during the elution process. Compared with traditional methods, this approach reduces impurity residues and eliminates the need for centrifugation. Furthermore, we compared the commonly used detection methods for *Panax* species identification, and the MIRA-CRISPR/Cas12a method demonstrates advantages in sensitivity, detection time, and cost, and does not require expensive equipment or trained personnel (Table S5). Both MIRA and CRISPR reactions operate at near-room temperature, and LFA detection requires minimal technical expertise. Collectively, these features make the system suitable for point-of-care testing (POCT) outside of laboratory settings. Furthermore, when integrated with portable electrochemical biosensors, CRISPR/Cas systems have the potential to achieve amplification-free nucleic acid detection [39]. This opens the possibility for real-time, on-site authentication of traditional Chinese medicines throughout the supply chain.

Currently, the MIRA-CRISPR/Cas12a platform developed in this study operates at near-room temperature, but its workflow still requires a two-step process. Future research will focus on simplifying the workflow into a "one-pot" reaction. Furthermore, this study is limited to distinguishing the two most common *Panax* species (PG and PQ) in the market. We attempted to include other *Panax*-related species. However, the absence of complete mitochondrial gene sequences in public databases has hindered accurate crRNA cross-reactivity prediction and experimental validation. Future work will involve sequencing these targets or selecting alternative genes to design species-specific crRNAs and implement multiplex detection strategies. For instance, in fluorescence detection, multiple Cas enzymes and fluorescent probes with different reporter groups can be used to detect multiple fluorescent signals within the corresponding wavelength ranges [42]. In LFA detection, lateral flow strips with dual test lines can be employed, which are matched with specifically labeled probes to enable naked-eye differentiation of two targets [35].

In CRISPR detection systems, the sensitivity of LFA is lower than that of fluorescence-based detection. LFA sensitivity is limited due to dilution of reaction products and reliance on visual interpretation [43]. Future improvements may include sample concentration, system volume optimization, and development of tools for band intensity quantification [21]. While the sensitivity of LFA detection is lower than that of fluorescence-based methods, its portability and instrument-free operation remain unparalleled [44,45].

Although the crRNAs designed in this study exhibited high specificity, there is still room for optimization to fully activate Cas12a cleavage activity. Despite evaluating multiple prediction algorithms, none demonstrated clear superiority. Further investigation into the influence of target sequence characteristics on Cas12/Cas13 trans-cleavage activity will be essential. The development of crRNA design algorithms tailored specifically for CRISPR-based nucleic acid detection platforms is highly anticipated.

## 5 Conclusion

In this study, we developed a novel, portable nucleic acid detection method based on MIRA-CRISPR/Cas12a technology, incorporating both fluorescence and lateral flow assay (LFA) readouts to distinguish *Panax ginseng* (PG) from *Panax quinquefolium* (PQ). This method demonstrated high sensitivity, specificity, and practical applicability, even in complex commercial samples and multi-herb formulations. Our findings highlight the potential of CRISPR/Cas-based detection systems in the field of traditional Chinese medicine (TCM) authentication and lay a solid foundation for their broader application in molecular identification and quality control of herbal products.

## Declaration of generative AI and AI-assisted technologies in the writing process

During the preparation of this work the authors used Grammarly in order to correct the gramm, spelling and clarity. After using this Grammarly, the authors reviewed and edited the content as needed and take full responsibility for the content of the publication.

## Funding

This research was funded by the Science and Technology Development Fund, Macau SAR (File no. 0075/2024/RIB2, 0105/2022/A2 and 006/2023/SKL).

## Declaration of competing interest

The authors state that there were no commercial or financial interests that might present a potential conflict of interest in this research.
