# Supplementary material for: Development of a MIRA-CRISPR/Cas12a-based nucleic acid detection system for the discrimination of Panax ginseng and Panax quinquefolium
Source: J Ginseng Res. 2025 Nov 28;50(2):100923. doi: 10.1016/j.jgr.2025.11.011 (PMC12959311; doi:10.1016/j.jgr.2025.11.011)
Supplement: Multimedia component 1 [file mmc1.docx]

**Development of a MIRA-CRISPR/Cas12a-Based Nucleic Acid Detection System for the Discrimination of *Panax ginseng* and *Panax quinquefolium***

Yanchao Yang^a, b^, Dongfan Yang ^a, b^, Meina Shi ^a, b^, Zifeng Huang ^a, b^, Xuening Zhang ^a, b^, Dayuan Zheng ^a, b^, Tong Chu ^a, b^, Wenzhe Ma^a, b,^ *

^a^State Key Laboratory of Mechanism and Quality of Chinese Medicine & Faculty of Chinese Medicine, Macau University of Science and Technology, Macau SAR, 999078, China

^b^Zhuhai MUST Science and Technology Research Institute, Macau University of Science and Technology, Hengqin Guangdong-Macao In-Depth Cooperation Zone, Guangdong, 519099, China

*Corresponding author. Tel.: +853-88972462, Fax: +853-28825886, Email: [wzma@must.edu.mo](mailto:wzma@must.edu.mo) (W.M.)

**Table S1 The sequence information of DNA and RNA**

| Name | Sequence (5‘-3’) |
| --- | --- |
| ITS2-F | ATGCGATACTTGGTGTGAAT |
| ITS3-R | GACGCTTCTCCAGACTACAAT |
| *nad4*-F | GGGCCGGAGGTACAGTAAAC |
| *nad4*-R | TGGAACCTCTCAACAGCTCC |
| MIRA-*nad4*-F1 | TGTATAGCACGAAAAACCTTTCGATACAAG |
| MIRA-*nad4*-F2 | AAAACCTTTCGATACAAGATAGGGCCGTTCA |
| MIRA-*nad4*-F3 | ATACAAGATAGGGCCGTTCACATGAAAGAAA |
| MIRA-*nad4*-F4 | GATAGGGCCGTTCACATGAAAGAAAGAAATT |
| MIRA-*nad4*-R1 | TACATAATGGTTCCTGCTTTCTACCCATAG |
| MIRA-*nad4*-R2 | TGCATAGGTACTCTTCGATGCAGCGGGGACG |
| MIRA-*nad4*-R3 | TGAACTGGAACATAGTGCATAGGTACTCTTC |
| MIRA-*nad4*-R4 | AACTGGAACATAGTGCATAGGTACTCTTCGA |
| T7-crRNA-F | GAAATTAATACGACTCACTATAGGT |
| T7-PG-crRNA-R | AACGTAGTAACTCGACTAGAATAATCTACACTTAGTAGAAATTACCTATAGTGAGTCGTATTAATTTC |
| T7-PQ-crRNA-R | TTGGGCCTACCTATCCCGATCCAATCTACACTTAGTAGAAATTACCTATAGTGAGTCGTATTAATTTC |
| crRNA-1 | AAUUUCUACUAAGUGUAGAUCCAGUCGAAAAGAGGGGUUCCUU |
| crRNA-2 | AAUUUCUACUAAGUGUAGAUUCCCUCUAUCAAAAUGAUCAAAA |
| crRNA-3 (PG-crRNA) | AAUUUCUACUAAGUGUAGAUUAUUCUAGUCGAGUUACUACGUU |
| crRNA-4 (PQ-crRNA) | AAUUUCUACUAAGUGUAGAUUGGAUCGGGAUAGGUAGGCCCAA |
| crRNA-5 | AAUUUCUACUAAGUGUAGAUGGUAUUUGGCCAAGUAUCCUACA |
| crRNA-6 | AAUUUCUACUAAGUGUAGAUGGUACCAAUUUUUGGGCCAAUUC |
| crRNA-7 | AAUUUCUACUAAGUGUAGAUCCAGACGAAAAGAGGGGUUCCUU |
| crRNA-8 | AAUUUCUACUAAGUGUAGAUUCGCUCUAUCAAAAUGAUCAAAA |

**Table S2 The information of 29 samples**

| No. | Sample | TCM classification | Source of purchase |
| --- | --- | --- | --- |
| S1 | *P. ginseng* | medicinal material | Online |
| S2 | *P. ginseng* | medicinal material | Online |
| S3 | *P. ginseng* | medicinal material | Online |
| S4 | *P. ginseng* | medicinal material | Online |
| S5 | *P. ginseng* | medicinal material | Online |
| S6 | *P. ginseng* | medicinal material | Online |
| S7 | *P. ginseng* | medicinal material | Herbal market |
| S8 | *P. ginseng* | medicinal material | Herbal market |
| S9 | *P. ginseng* | medicinal material | Online |
| S10 | *P. ginseng* | herbal piece | Pharmacy |
| S11 | *P. ginseng* | herbal piece | Pharmacy |
| S12 | *P. ginseng* | herbal piece | Online |
| S13 | *P. ginseng* | patent medicine | Online |
| S14 | *P. ginseng* | patent medicine | Online |
| S15 | *P. ginseng* | patent medicine | Online |
| S16 | *P. quinquefolium* | medicinal material | Online |
| S17 | *P. quinquefolium* | medicinal material | Online |
| S18 | *P. quinquefolium* | medicinal material | Online |
| S19 | *P. quinquefolium* | herbal piece | Online |
| S20 | *P. quinquefolium* | herbal piece | Online |
| S21 | *P. quinquefolium* | herbal piece | Online |
| S22 | *P. quinquefolium* | herbal piece | Online |
| S23 | *P. quinquefolium* | herbal piece | Online |
| S24 | *P. quinquefolium* | herbal piece | Pharmacy |
| S25 | *P. quinquefolium* | herbal piece | Pharmacy |
| S26 | *P. quinquefolium* | herbal piece | Pharmacy |
| S27 | *P. quinquefolium* | herbal piece | Pharmacy |
| S28 | *P. quinquefolium* | herbal piece | Pharmacy |
| S29 | *P. quinquefolium* | patent medicine | Online |

**Table S3 The sequencing report for the ITS2 genes of 26 samples**

| No. | Sequence (5’-3’) |
| --- | --- |
| S1 | CGCATCGCGTCGCCCCCCAACCCATCACTCCCTTGCGGGAGTTGAGGCGGAGGGGCGGATAATGGCCTCCCGTGTCTCACCGCGCGGTTGGCCCAAATGCGAGTCCTTGGCGATGGACGTCACGACAAGTGGTGGTTGTAAAAAGCCCTCTTCTCATGTCGTGCGGTGACCCGTCGCCAGCAAAAGCTCTCATGACCCTGTTGCGCCGTCCTCGACGTGCGCTCCGACCG |
| S2 | CGCATCGCGTCGCCCCCCAACCCATCACTCCTTTGCGGGAGTCGAGGCGGAGGGGCGGATAATGGCCTCCCGTGTCTCACCGCGCGGTTGGCCCAAATGCGAGTCCTTGGCGATGGACGTCACGACAAGTGGTGGTTGTAAAAAGCCCTCTTCTCATGTCGTGCGGTGACCCGTCGCCAGCAAAAGCTCTCATGACCCTGTTGCGCCGTCCTCGACGTGCGCTCCGACCG |
| S3 | CGCATCGCGTCGCCCCCCAACCCATCACTCCCTTGCGGGAGTTGAGGCGGAGGGGCGGATAATGGCCTCCCGTGTCTCACCGCGCGGTTGGCCCAAATGCGAGTCCTTGGCGATGGACGTCACGACAAGTGGTGGTTGTAAAAAGCCCTCTTCTCATGTCGTGCGGTGACCCGTCGCCAGCAAAAGCTCTCATGACCCTGTTGCGCCGTCCTCGACGTGCGCTCCGACCG |
| S4 | CGCATCGCGTCGCCCCCCAACCCATCACTCCCTTGCGGGAGTTGAGGCGGAGGGGCGGATAATGGCCTCCCGTGTCTCACCGCGCGGTTGGCCCAAATGCGAGTCCTTGGCGATGGACGTCACGACAAGTGGTGGTTGTAAAAAGCCCTCTTCTCATGTCGTGCGGTGACCCGTCGCCAGCAAAAGCTCTCATGACCCTGTTGCGCCGTCCTCGACGTGCGCTCCGACCG |
| S5 | CGCATCGCGTCGCCCCCCAACCCATCACTCCCTTGCGGGAGTTGAGGCGGAGGGGCGGATAATGGCCTCCCGTGTCTCACCGCGCGGTTGGCCCAAATGCGAGTCCTTGGCGATGGACGTCACGACAAGTGGTGGTTGTAAAAAGCCCTCTTCTCATGTCGTGCGGTGACCCGTCGCCAGCAAAAGCTCTCATGACCCTGTTGCGCCGTCCTCGACGTGCGCTCCGACCG |
| S6 | CGCATCGCGTCGCCCCCCAACCCATCACTCCCTTGCGGGAGTTGAGGCGGAGGGGCGGATAATGGCCTCCCGTGTCTCACCGCGCGGTTGGCCCAAATGCGAGTCCTTGGCGATGGACGTCACGACAAGTGGTGGTTGTAAAAAGCCCTCTTCTCATGTCGTGCGGTGACCCGTCGCCAGCAAAAGCTCTCATGACCCTGTTGCGCCGTCCTCGACGTGCGCTCCGACCG |
| S7 | CGCATCGCGTCGCCCCCCAACCCATCACTCCCTTGCGGGAGTTGAGGCGGAGGGGCGGATAATGGCCTCCCGTGTCTCACCGCGCGGTTGGCCCAAATGCGAGTCCTTGGCGATGGACGTCACGACAAGTGGTGGTTGTAAAAAGCCCTCTTCTCATGTCGTGCGGTGACCCGTCGCCAGCAAAAGCTCTCATGACCCTGTTGCGCCGTCCTCGACGTGCGCTCCGACCG |
| S8 | CGCATCGCGTCGCCCCCCAACCCATCACTCCCTTGCGGGAGTTGAGGCGGAGGGGCGGATAATGGCCTCCCGTGTCTCACCGCGCGGTTGGCCCAAATGCGAGTCCTTGGCGATGGACGTCACGACAAGTGGTGGTTGTAAAAAGCCCTCTTCTCATGTCGTGCGGTGACCCGTCGCCAGCAAAAGCTCTCATGACCCTGTTGCGCCGTCCTCGACGTGCGCTCCGACCG |
| S9 | CGCATCGCGTCGCCCCCCAACCCATCACTCCCTTGCGGGAGTTGAGGCGGAGGGGCGGATAATGGCCTCCCGTGTCTCACCGCGCGGTTGGCCCAAATGCGAGTCCTTGGCGATGGACGTCACGACAAGTGGTGGTTGTAAAAAGCCCTCTTCTCATGTCGTGCGGTGACCCGTCGCCAGCAAAAGCTCTCATGACCCTGTTGCGCCGTCCTCGACGTGCGCTCCGACCG |
| S10 | CGCATCGCGTCGCCCCCCAACCCATCACTCCCTTGCGGGAGTTGAGGCGGAGGGGCGGATAATGGCCTCCCGTGTCTCACCGCGCGGTTGGCCCAAATGCGAGTCCTTGGCGATGGACGTCACGACAAGTGGTGGTTGTAAAAAGCCCTCTTCTCATGTCGTGCGGTGACCCGTCGCCAGCAAAAGCTCTCATGACCCTGTTGCGCCGTCCTCGACGTGCGCTCCGACCG |
| S11 | CGCATCGCGTCGCCCCCCAACCCATCACTCCCTTGCGGGAGTTGAGGCGGAGGGGCGGATAATGGCCTCCCGTGTCTCACCGCGCGGTTGGCCCAAATGCGAGTCCTTGGCGATGGACGTCACGACAAGTGGTGGTTGTAAAAAGCCCTCTTCTCATGTCGTGCGGTGACCCGTCGCCAGCAAAAGCTCTCATGACCCTGTTGCGCCGTCCTCGACGTGCGCTCCGACCG |
| S12 | CGCATCGCGTCGCCCCCCAACCCATCACTCCCTTGCGGGAGTTGAGGCGGAGGGGCGGATAATGGCCTCCCGTGTCTCACCGCGCGGTTGGCCCAAATGCGAGTCCTTGGCGATGGACGTCACGACAAGTGGTGGTTGTAAAAAGCCCTCTTCTCATGTCGTGCGGTGACCCGTCGCCAGCAAAAGCTCTCATGACCCTGTTGCGCCGTCCTCGACGTGCGCTCCGACCG |
| S16 | CGCATCGCGTCGCCCCCCAACCCATCACTCCTTTGCGGGAGTCGAGGCGGAGGGGCGGATAATGGCCTCCCGTGTCTCACCGCGCGGTTGGCCCAAATGCGAGTCCTTGGCGATGGACGTCACGACAAGTGGTGGTTGTAAAAAGCCCTCTTCTCATGTCGTGCGGTGACCCGTCGCCAGCAAAAGCTCTCATGACCCTGTTGCGCCGTCCTCGACGTGCGCTCCGACCG |
| S17 | CGCATCGCGTCGCCCCCCAACCCATCACTCCTTTGCGGGAGTCGAGGCGGAGGGGCGGATAATGGCCTCCCGTGTCTCACCGCGCGGTTGGCCCAAATGCGAGTCCTTGGCGATGGACGTCACGACAAGTGGTGGTTGTAAAAAGCCCTCTTCTCATGTCGTGCGGTGACCCGTCGCCAGCAAAAGCTCTCATGACCCTGTTGCGCCGTCCTCGACGTGCGCTCCGACCG |
| S18 | CGCATCGCGTCGCCCCCCAACCCATCACTCCTTTGCGGGAGTCGAGGCGGAGGGGCGGATAATGGCCTCCCGTGTCTCACCGCGCGGTTGGCCCAAATGCGAGTCCTTGGCGATGGACGTCACGACAAGTGGTGGTTGTAAAAAGCCCTCTTCTCATGTCGTGCGGTGACCCGTCGCCAGCAAAAGCTCTCATGACCCTGTTGCGCCGTCCTCGACGTGCGCTCCGACCG |
| S19 | CGCATCGCGTCGCCCCCCAACCCATCACTCCTTTGCGGGAGTCGAGGCGGAGGGGCGGATAATGGCCTCCCGTGTCTCACCGCGCGGTTGGCCCAAATGCGAGTCCTTGGCGATGGACGTCACGACAAGTGGTGGTTGTAAAAAGCCCTCTTCTCATGTCGTGCGGTGACCCGTCGCCAGCAAAAGCTCTCATGACCCTGTTGCGCCGTCCTCGACGTGCGCTCCGACCG |
| S20 | CGCATCGCGTCGCCCCCCAACCCATCACTCCTTTGCGGGAGTCGAGGCGGAGGGGCGGATAATGGCCTCCCGTGTCTCACCGCGCGGTTGGCCCAAATGCGAGTCCTTGGCGATGGACGTCACGACAAGTGGTGGTTGTAAAAAGCCCTCTTCTCATGTCGTGCGGTGACCCGTCGCCAGCAAAAGCTCTCATGACCCTGTTGCGCCGTCCTCGACGTGCGCTCCGACCG |
| S21 | CGCATCGCGTCGCCCCCCAACCCATCACTCCTTTGCGGGAGTCGAGGCGGAGGGGCGGATAATGGCCTCCCGTGTCTCACCGCGCGGTTGGCCCAAATGCGAGTCCTTGGCGATGGACGTCACGACAAGTGGTGGTTGTAAAAAGCCCTCTTCTCATGTCGTGCGGTGACCCGTCGCCAGCAAAAGCTCTCATGACCCTGTTGCGCCGTCCTCGACGTGCGCTCCGACCG |
| S22 | CGCATCGCGTCGCCCCCCAACCCATCACTCCTTTGCGGGAGTCGAGGCGGAGGGGCGGATAATGGCCTCCCGTGTCTCACCGCGCGGTTGGCCCAAATGCGAGTCCTTGGCGATGGACGTCACGACAAGTGGTGGTTGTAAAAAGCCCTCTTCTCATGTCGTGCGGTGACCCGTCGCCAGCAAAAGCTCTCATGACCCTGTTGCGCCGTCCTCGACGTGCGCTCCGACCG |
| S23 | CGCATCGCGTCGCCCCCCAACCCATCACTCCTTTGCGGGAGTCGAGGCGGAGGGGCGGATAATGGCCTCCCGTGTCTCACCGCGCGGTTGGCCCAAATGCGAGTCCTTGGCGATGGACGTCACGACAAGTGGTGGTTGTAAAAAGCCCTCTTCTCATGTCGTGCGGTGACCCGTCGCCAGCAAAAGCTCTCATGACCCTGTTGCGCCGTCCTCGACGTGCGCTCCGACCG |
| S24 | CGCATCGCGTCGCCCCCCAACCCATCACTCCTTTGCGGGAGTCGAGGCGGAGGGGCGGATAATGGCCTCCCGTGTCTCACCGCGCGGTTGGCCCAAATGCGAGTCCTTGGCGATGGACGTCACGACAAGTGGTGGTTGTAAAAAGCCCTCTTCTCATGTCGTGCGGTGACCCGTCGCCAGCAAAAGCTCTCATGACCCTGTTGCGCCGTCCTCGACGTGCGCTCCGACCG |
| S25 | CGCATCGCGTCGCCCCCCAACCCATCACTCCTTTGCGGGAGTCGAGGCGGAGGGGCGGATAATGGCCTCCCGTGTCTCACCGCGCGGTTGGCCCAAATGCGAGTCCTTGGCGATGGACGTCACGACAAGTGGTGGTTGTAAAAAGCCCTCTTCTCATGTCGTGCGGTGACCCGTCGCCAGCAAAAGCTCTCATGACCCTGTTGCGCCGTCCTCGACGTGCGCTCCGACCG |
| S26 | CGCATCGCGTCGCCCCCCAACCCATCACTCCTTTGCGGGAGTCGAGGCGGAGGGGCGGATAATGGCCTCCCGTGTCTCACCGCGCGGTTGGCCCAAATGCGAGTCCTTGGCGATGGACGTCACGACAAGTGGTGGTTGTAAAAAGCCCTCTTCTCATGTCGTGCGGTGACCCGTCGCCAGCAAAAGCTCTCATGACCCTGTTGCGCCGTCCTCGACGTGCGCTCCGACCG |
| S27 | CGCATCGCGTCGCCCCCCAACCCATCACTCCTTTGCGGGAGTCGAGGCGGAGGGGCGGATAATGGCCTCCCGTGTCTCACCGCGCGGTTGGCCCAAATGCGAGTCCTTGGCGATGGACGTCACGACAAGTGGTGGTTGTAAAAAGCCCTCTTCTCATGTCGTGCGGTGACCCGTCGCCAGCAAAAGCTCTCATGACCCTGTTGCGCCGTCCTCGACGTGCGCTCCGACCG |
| S28 | CGCATCGCGTCGCCCCCCAACCCATCACTCCTTTGCGGGAGTCGAGGCGGAGGGGCGGATAATGGCCTCCCGTGTCTCACCGCGCGGTTGGCCCAAATGCGAGTCCTTGGCGATGGACGTCACGACAAGTGGTGGTTGTAAAAAGCCCTCTTCTCATGTCGTGCGGTGACCCGTCGCCAGCAAAAGCTCTCATGACCCTGTTGCGCCGTCCTCGACGTGCGCTCCGACCG |
| S29 | CGCATCGCGTCGCCCCCCAACCCATCACTCCTTTGCGGGAGTCGAGGCGGAGGGGCGGATAATGGCCTCCCGTGTCTCACCGCGCGGTTGGCCCAAATGCGAGTCCTTGGCGATGGACGTCACGACAAGTGGTGGTTGTAAAAAGCCCTCTTCTCATGTCGTGCGGTGACCCGTCGCCAGCAAAAGCTCTCATGACCCTGTTGCGCCGTCCTCGACGTGCGCTCCGACCG |

**Table S4 The information of Chinese patent medicines**

| Sample | Name | Composition |
| --- | --- | --- |
| S13 | Renshenjianpiwan | *Panax ginseng, Atractylodes macrocephala, Poria cocos, Dioscorea opposita , Citrus reticulata Blanco, Aucklandia lappa, Amomum villosum, Astragalus membranaceus, Angelica sinensis, Ziziphus jujuba, Polygala tenuifolia* |
| S14 | Shenlingbaizhusan | *Panax ginseng, Poria cocos, Atractylodes macrocephala, Dioscorea opposita , Lablab purpureus, Nelumbo nuciferaGaertn, Coix lacryma-jobi, Amomum villosum, Platycodon grandifloras, Glycyrrhiza uralensis* |
| S15 | Wujibaifengwan | *Gallus gallus domesticus, Panax ginseng, Paeonia lactiflora, Salvia miltiorrhiza, Cyperus rotundus, Angelica sinensis, Ostreae Concha, Cornu Cervi, Ootheca Mantidis, Glycyrrhiza uralensis, Artemisia annua, Asparagus cochinchinensis, Rehmannia glutinosa, Ligusticum chuanxiong, Astragalus membranaceus, Stellaria dichotoma, Euryale ferox, Dioscorea opposite, Mel* |
| S29 | Yangshen capsules | *Panax quinquefolium* |

**Table S5 Comparison of characteristics of common detection methods for *Panax* species identification**

| Method | Sensitivity | Detection time | Cost per sample | Equipment needed | Professional operators | Reference |
| --- | --- | --- | --- | --- | --- | --- |
| MIRA-CRISPR/Cas12a (Fluorescence) | 10⁻⁴ ng/μL | ~1 h | low | Microplate reader, heating block | No | This study |
| MIRA-CRISPR/Cas12a (LFA) | 10⁻³ ng/μL | ~1 h | low | Heating block | No | This study |
| DNA Barcoding | Comparable | ~24–48 h | Moderate | Thermal cycler, sequencer, gel imaging system | Yes | [1] |
| HPLC-MS | Relatively low | ~4–6 h | High | HPLC-MS system, sample pretreatment equipment | Yes | [2] |
| UPLC/QTOF-MS | Relatively low | ~4–6 h | High | UPLC/QTOF-MS system | Yes | [3] |

**
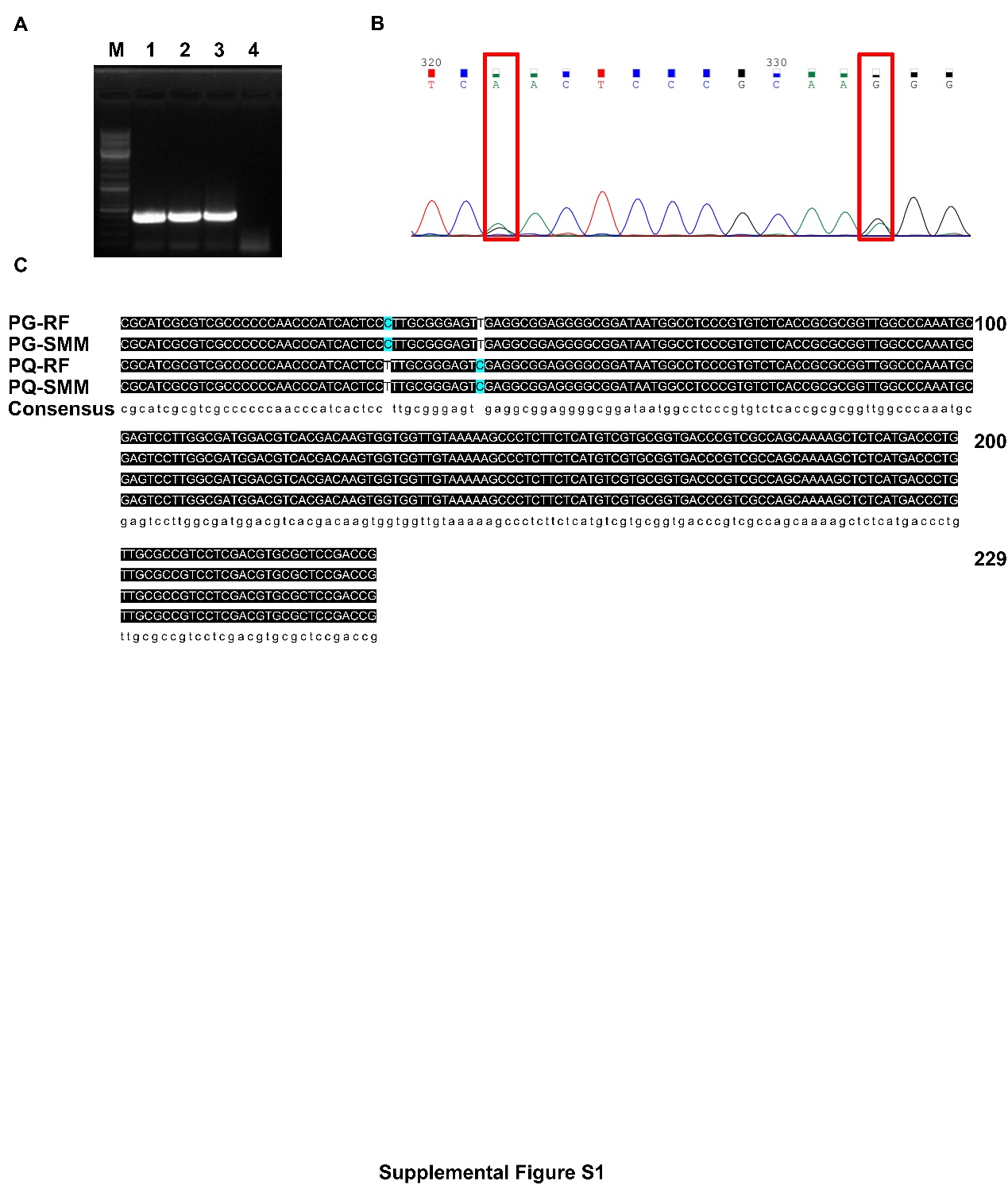
**

**Supplemental Figure S1. DNA barcoding analysis of PG and PQ standard medicinal materials.** A. Agarose gel image of PCR products from PG and PQ standard medicinal materials and their mixture. M: Marker 1: PG standard medicinal material (PG-SMM) 2: PQ standard medicinal material (PQ-SMM) 3: The mixture of PG-SMM and PQ-SMM 4: no template control (NTC). B. The partial sequencing chromatogram of PG-SMM and PQ-SMM mixture. C. Alignment of ITS2 sequencing sequences of PG-SMM and PQ-SMM with PG reference sequence (PG-RF) and PQ reference sequence (PQ-RF).


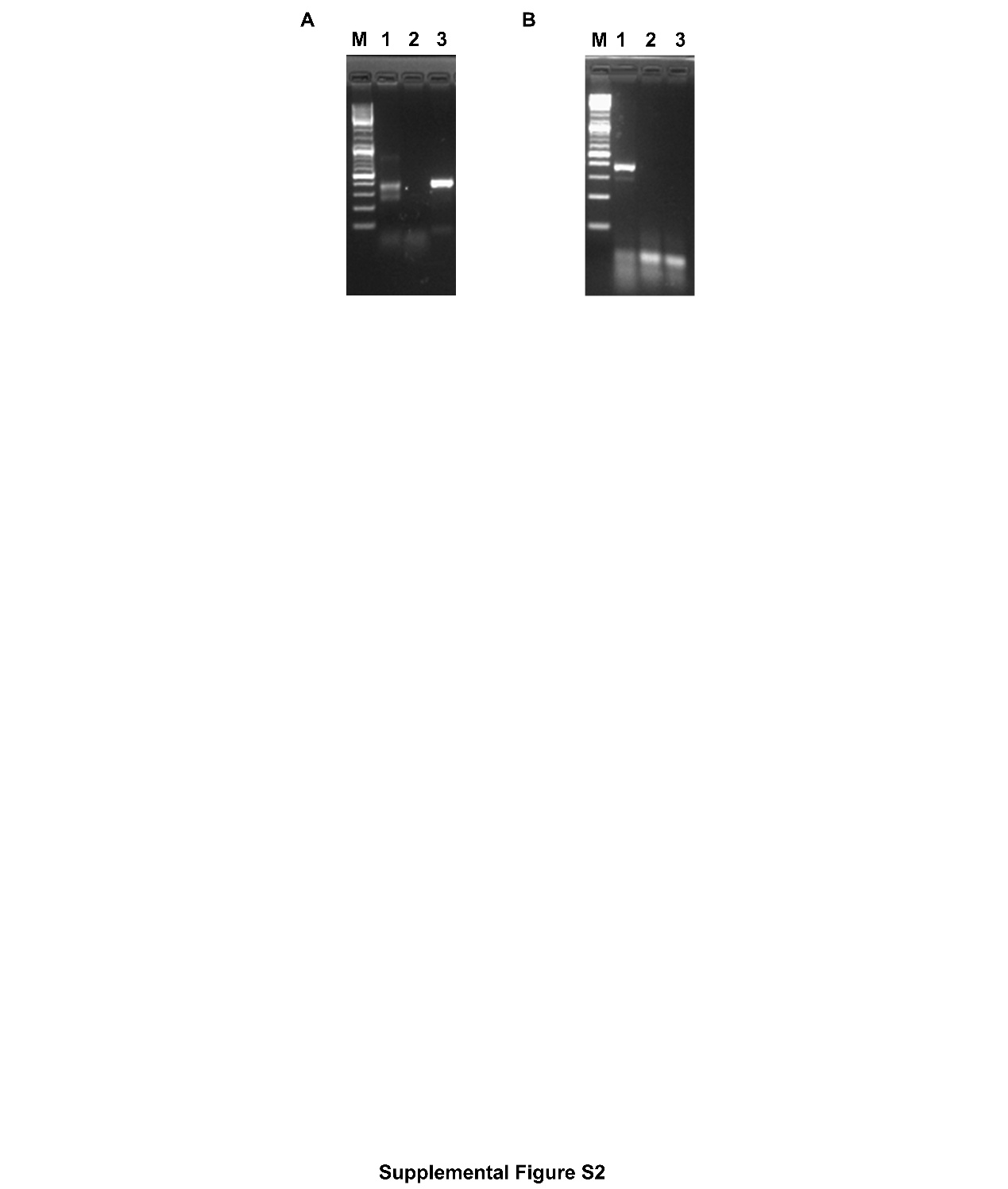


**Supplemental Figure S2. Cas12a cis- and trans-cleavage activity assays** A. Cis-cleavage activity of Cas12a. M: Marker 1: Cas12a + crRNA + target DNA 2: Cas12a + crRNA 3: target DNA. B. Trans-cleavage activity of Cas12a. M: Marker 1: Cas12a + crRNA + target DNA + 68 bp ssDNA 2: Cas12a + crRNA + 68 bp ssDNA 3: 68 bp ssDNA.


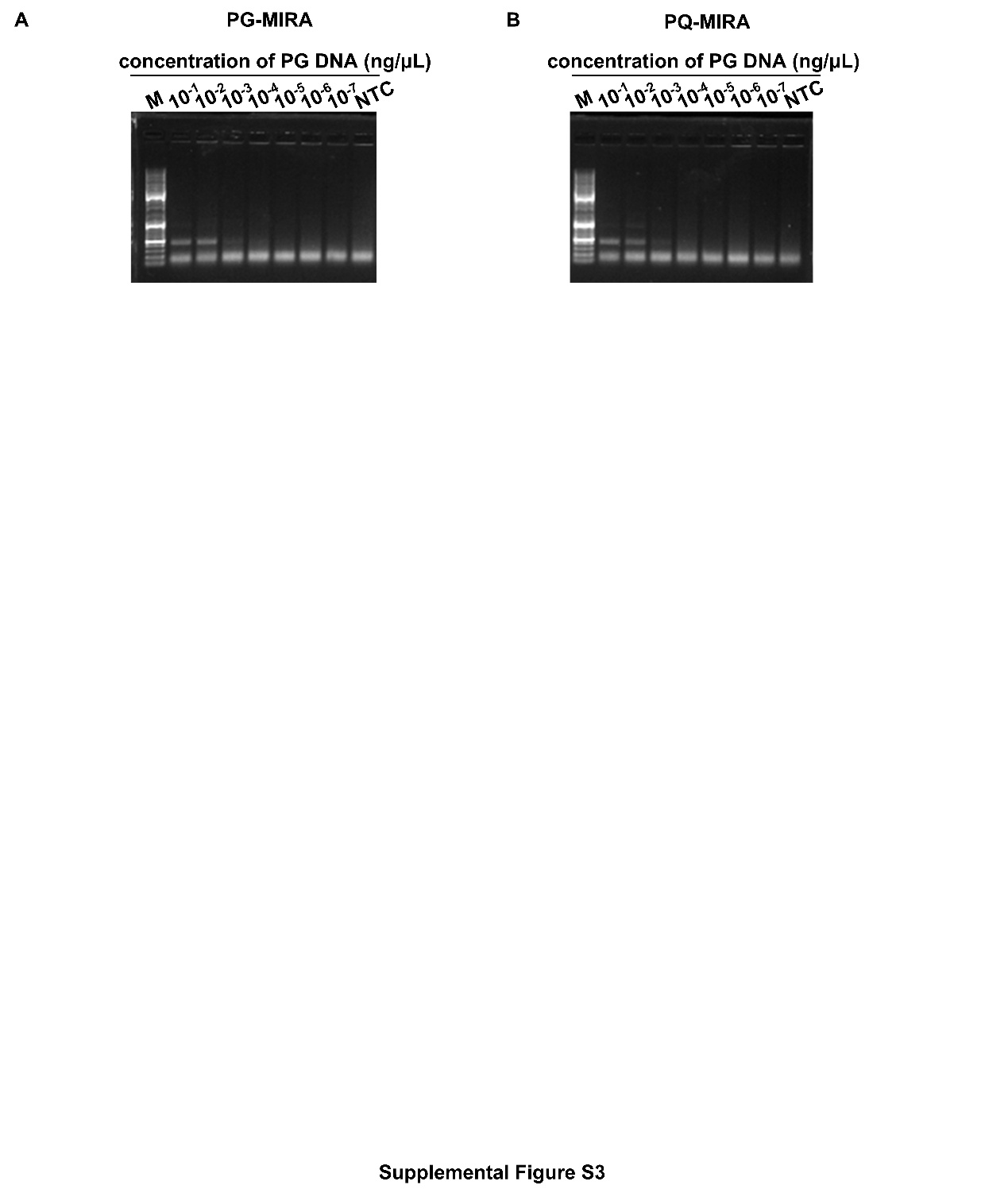


**Supplemental Figure S3. Sensitivity assessment based on MIRA of PG-SMM and PQ-SMM.** A-B. Agarose gel image of MIRA of 10-fold diluted DNA templates of PG-SMM and PQ-SMM (from 10^-7^ to 10^-1^ ng/µL). M: Marker.


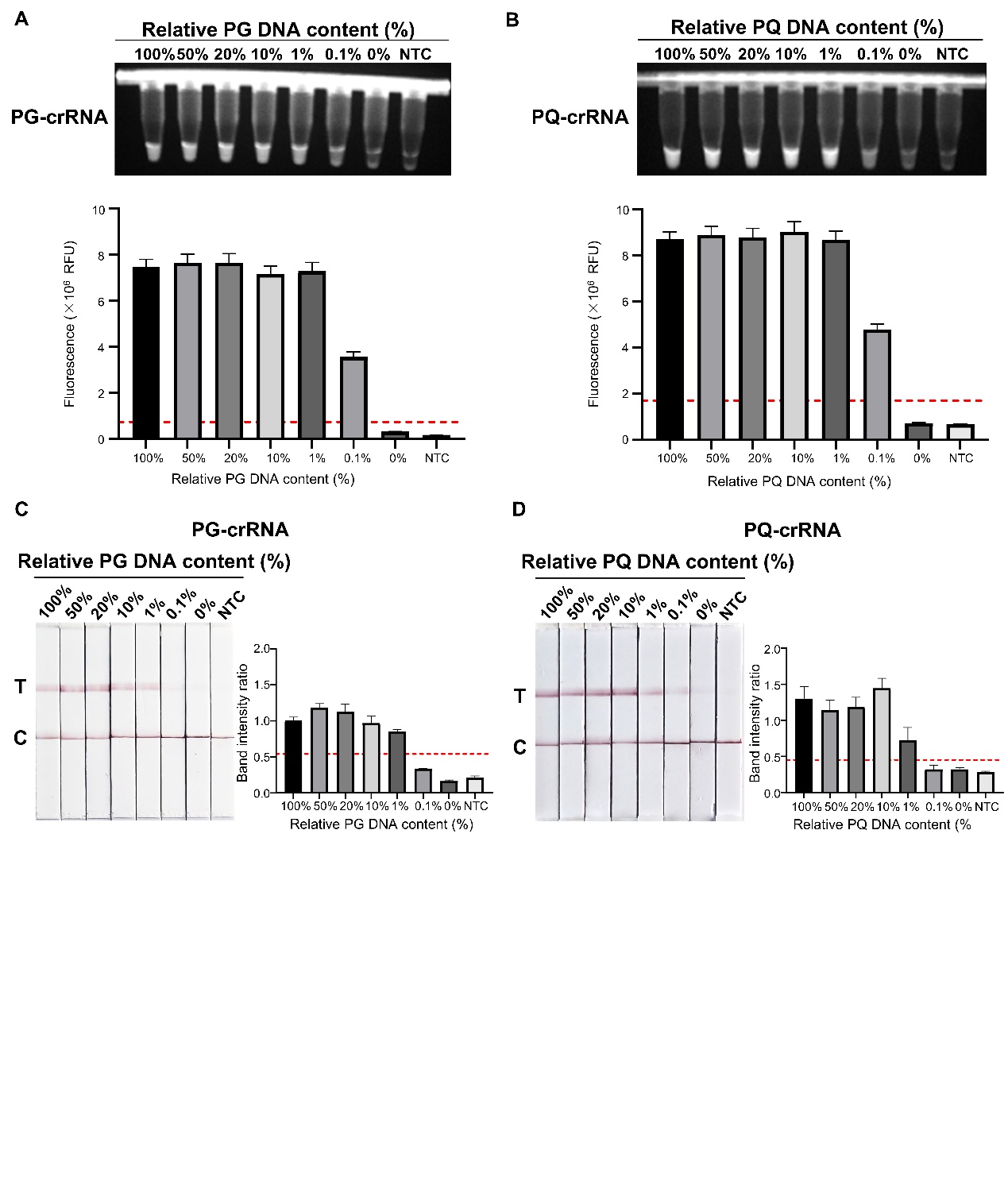


**Supplemental Figure S4.** **Detectability assessment based on MIRA-CRISPR/Cas12a detection of PG-SMM and PQ-SMM.** A-B. The images of fluorescence detection and fluorescence signals of MIRA-CRISPR/Cas12a-based detection of different proportions (100%, 50%, 20%, 10%, 1%, 0.1%, 0%) of PG-SMM and PQ-SMM DNA with PG-crRNA and PQ-crRNA (the cutoff fluorescence intensity values for PG-crRNA and for PQ-crRNA at 7.4 × 10⁵ and 1.7 × 10⁶ RFU for positive samples). C-D. The images of lateral flow assay (LFA) detection and band intensity ratios of MIRA-CRISPR/Cas12a-based detection of different proportions (100%, 50%, 20%, 10%, 1%, 0.1%, 0%) of PG-SMM and PQ-SMM DNA with PG-crRNA and PQ-crRNA (the cutoff LFA band intensity ratios for PG-crRNA and for PQ-crRNA at 0.54 and 0.45 for positive samples). C: control line T: test line. All data are presented as the mean ± SD (n = 3 technical replicates). All experiments were performed at least three biologically independent times with similar results.


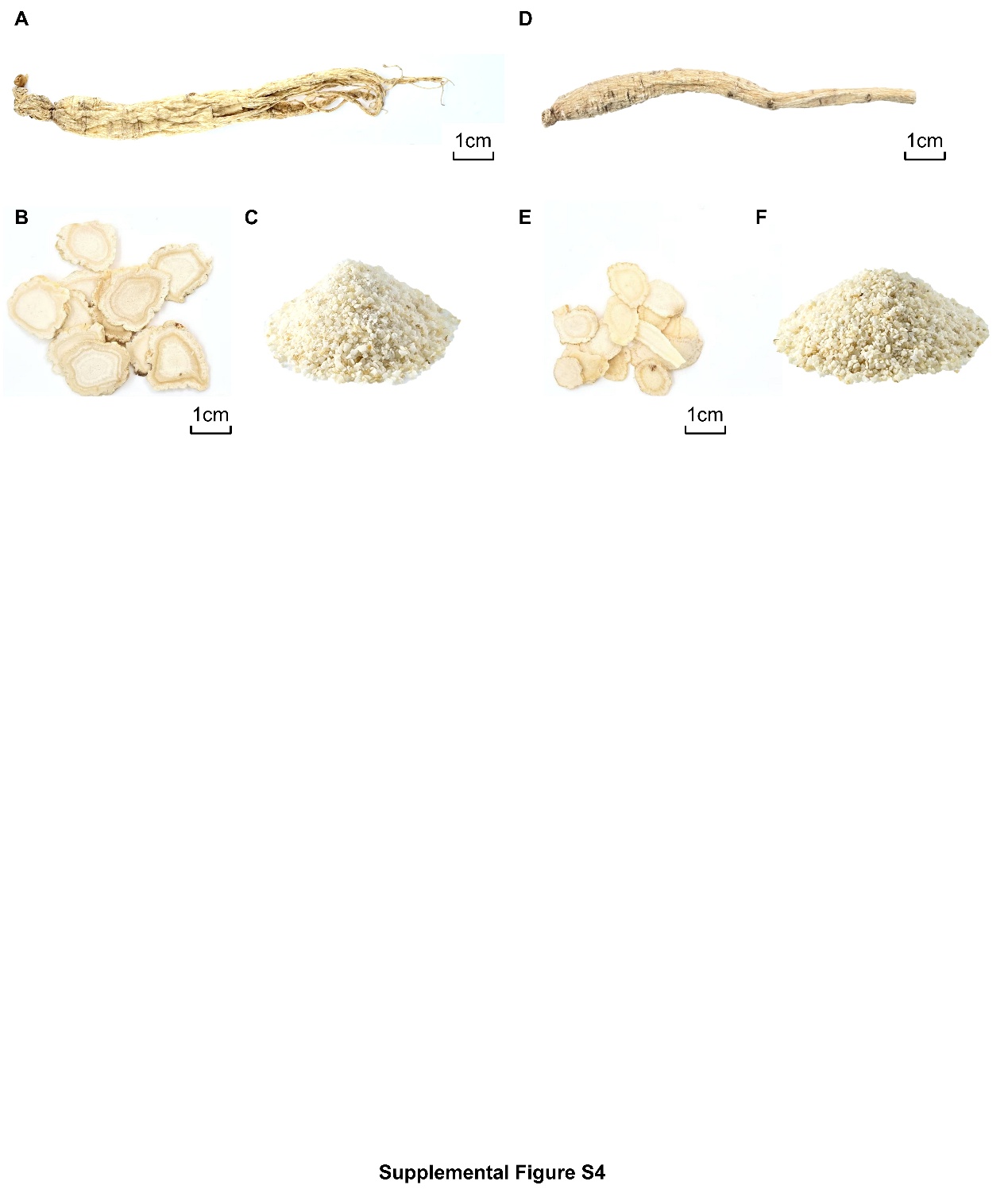


**Supplemental Figure S5. Morphological characteristics of different traditional Chinese medicine classification of *Panax ginseng* (PG) and *Panax quinquefolium*  (PQ).** A-C. The medicinal material, herbal pieces and powders of PG. D-F. The medicinal material, herbal pieces and powders of PQ (scale bar = 1 cm).

**References**

[1] Chen, X., B. Liao, J. Song, X. Pang, J. Han, and S. Chen, A fast SNP identification and analysis of intraspecific variation in the medicinal Panax species based on DNA barcoding. Gene, 2013. 530(1): p. 39-43.

[2] Yang, W., X. Qiao, K. Li, J. Fan, T. Bo, D.-a. Guo, and M. Ye, Identification and differentiation of Panax ginseng, Panax quinquefolium, and Panax notoginseng by monitoring multiple diagnostic chemical markers. Acta Pharmaceutica Sinica B, 2016. 6(6): p. 568-575.

[3] Lee, J.W., B.-R. Choi, Y.-C. Kim, D.J. Choi, Y.-S. Lee, G.-S. Kim, N.-I. Baek, S.-Y. Kim, and D.Y. Lee, Comprehensive Profiling and Quantification of Ginsenosides in the Root, Stem, Leaf, and Berry of Panax ginseng by UPLC-QTOF/MS. Molecules, 2017. 22(12): p. 2147.
